# Synthesis and Diameter-dependent Thermal Conductivity of InAs Nanowires

**DOI:** 10.1007/s40820-014-0002-8

**Published:** 2014-09-19

**Authors:** Pinyun Ren, Xiaoli Zhu, Jinyun Han, Jinyou Xu, Liang Ma, Honglai Li, Xiujuan Zhuang, Hong Zhou, Qinglin Zhang, Minggang Xia, Anlian Pan

**Affiliations:** 1 Key Laboratory for Micro-Nano Physics and Technology of Hunan Province, State Key Laboratory of Chemo/Biosensing and Chemometrics, College of Physics and Microelectronics Science, Hunan University, Changsha, 410082 China; 2 Condensed Matter Physics Laboratory, Center on Experimental Physics, School of Science, Xi’an Jiaotong University, Xi’an, 710049 Shaanxi China

**Keywords:** InAs nanowires, Chemical vapor deposition, Thermal conductivity, Phonon-boundary scattering

## Abstract

In this work, we synthesized high-quality InAs nanowires by a convenient chemical vapor deposition method, and developed a simple laser heating method to measure the thermal conductivity of a single InAs nanowire in air. During the measurement, a focused laser was used to heat one end of a freely suspended nanowire, with its other end embedded into a carbon conductive adhesive. In order to obtain the thermal conductivity of InAs nanowires, the heat loss in the heat transfer process was estimated, which includes the heat loss through air conduction, the heat convection, and the radiation loss. The absorption ratio of the laser power in the InAs nanowire was calculated. The result shows that the thermal conductivity of InAs nanowires monotonically increases from 6.4 W m^−1^ K^−1^ to 10.5 W m^−1^ K^−1^ with diameters increasing from 100 nm to 190 nm, which is ascribed to the enhanced phonon-boundary scattering.

## Introduction

In recent years, materials with high electrical and low thermal conductivity have attracted considerable attention due to their potential applications in thermoelectric devices [1–3]. In addition, it has been demonstrated that the thermoelectric property of material is not only composition dependent but also morphology dependent [4–7]. Nanowires (NWs) are promising candidates for thermoelectric devices due to the low thermal conductivity and high electronic mobility [2, 8–10]. As an important III–V semiconductor, InAs NWs have been extensively studied for nanoelectronics due to its high electron mobility [11, 12]. At the same time, its thermal property has drawn a wide attention. For example, Feng et al. have comprehensively studied the thermal conductivity of wurtzite and zinc blende InAs NWs [13]. Annl et al. reported that the thermal conductivity of InAs NW could be significantly reduced by embedding polymethylmethacrylate (PMMA) in InAs NW array [14]. Yuan et al. realized the gate-controlled thermoelectric properties of InAs NWs [15]. However, the characterization of thermal conductivity in air has not been reported for InAs NWs, which is particularly important for applications in thermoelectric device.

In this work, we successfully synthesized high-quality InAs NWs through a convenient CVD process [16–18], and systematically studied the diameter-dependent thermal conductivity of InAs NWs in air by a simple laser heating method. The obtained thermal conductivity of InAs NWs lies in the range of 6.4–10.5 W m^−1^K^−1^ depending on their diameters, and presents a monotonic increase with increasing diameter, which agrees well with the previously reported results.

## Experimental Part

### Synthesis and Characterization of InAs NWs

**Fig. 1:**
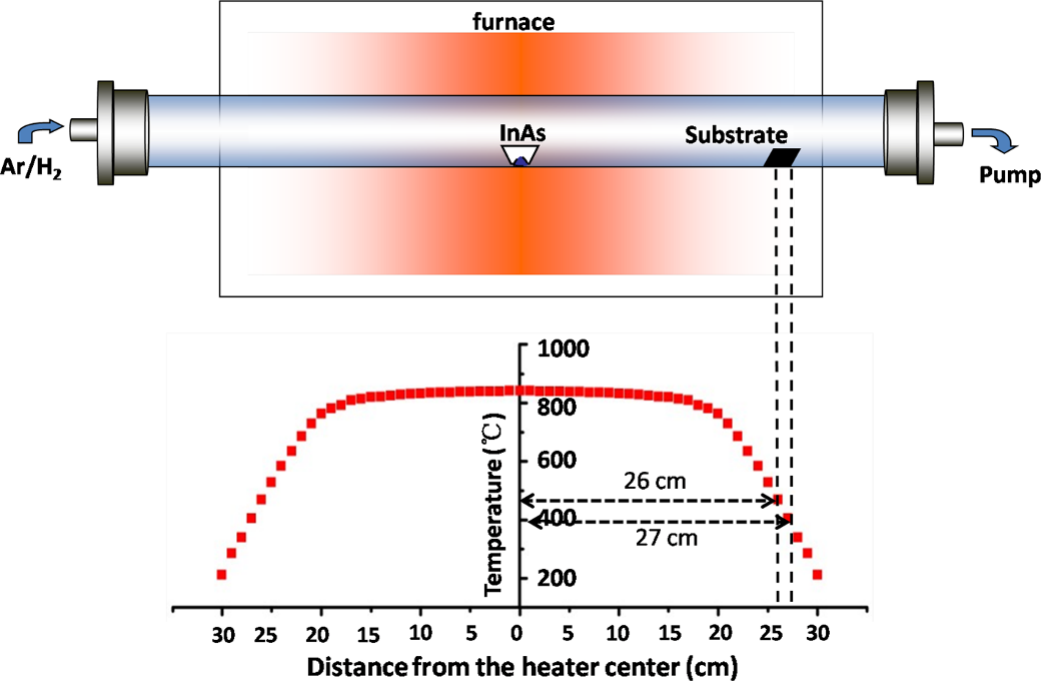
Schematic diagram of the experimental setup and growth conditions

The InAs NWs were synthesized by a convenient chemical vapor deposition (CVD) method. The schematic diagram of the experimental setup and growth conditions is shown in Fig. 1. Typically, an alumina boat with InAs (99.99 % purity, Alfa Aesar) powder was firstly placed at the center of a quartz tube. Two Si wafers coated with 2-nm-thick gold films were placed at the downstream of the gas flow (25–27 cm away from the center of tube furnace) for sample deposition. Argon–hydrogen (95 % Ar and 5 % H_2_) mixture was introduced into the quartz tube with a constant flowing rate (40 SCCM). The furnace was then heated to 850° in 30 min while maintaining the pressure at 2 mbar. After 30 min of growth, the furnace was naturally cooled down to room temperature. Figure 1 shows the temperature profile of tube furnace that has been measured with thermocouples.

**Fig. 2:**
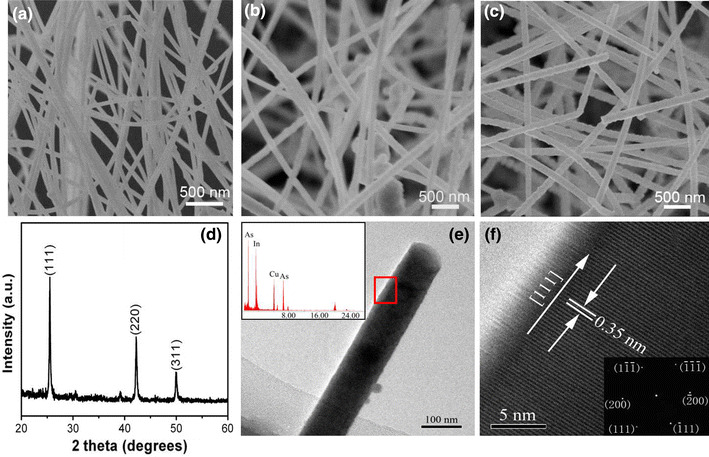
**a**–**c** SEM images of NWs collected from 400°, 440°, and 460° deposited zone, respectively; **d** XRD of as-prepared InAs NWs; **e** Low-magnification TEM images of single NW and corresponding in situ EDS spectrum; **f** Lattice-resolved high-magnification TEM image and corresponding SAED

As we all know, thermal conductivity not only correlates with material composition, but also depends on crystal structure [19, 20]. In this regard, the composition and structure of NWs were firstly characterized. Experimental results indicated that the InAs NWs were only deposited in downstream location 26–27 cm away from the center of the tube furnace, corresponding to temperature ranging from ~460 to ~400 °C. The diameters of NWs increased from 100 to 190 nm with the increase of the deposited temperature, as can be seen from SEM images in Fig. 2a–c. The XRD pattern of the product is shown in Fig. 2d, which can be indexed into the cubic phase InAs (JCPDS NO. 89-3314). The strong and narrow diffraction peaks demonstrate its good crystallinity. Figure 2e shows a bright-field TEM image and its corresponding in situ EDX spectra of a single NW. Besides the peaks of Cu from micro-grid, the only presence of In and As peaks indicates the formation of pure InAs NWs. Figure 2f displays the corresponding high-resolution TEM (HRTEM) image of the NWs. The measured adjacent plane spacing along the growth direction is 0.35 nm, corresponding to the (111) lattice plane of cubic InAs. The single crystalline nature of the InAs NW is further confirmed by selected area electron diffraction (SAED) pattern in the inset, which presents only one set of diffraction spots.

### Test of Thermal Conductivity

**Fig. 3:**
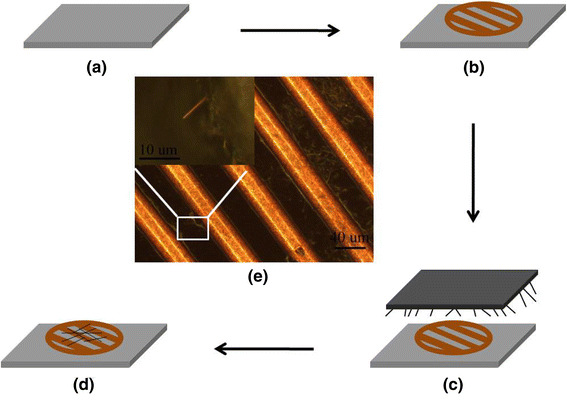
**a**–**d** The schematic illustration of sample preparation for thermal conductivity test; **e** Optical image of prepared sample

At the moment, three-omega method is the most common technique for measurement of the thermal conductivity of NW [21, 22]. Here, we developed a simple laser heating method to measure the thermal conductivity of a single InAs NW in air. The sample preparation for thermal conductivity measurement is schematically shown in Fig. 3a–d. A carbon conductive adhesive film (CAF) with 1 centimeter in length and width was firstly prepared (Fig. 3a). A micro-grid used for TEM test was then placed onto the CAF (Fig. 3b). Then the Si wafer deposited with InAs NWs was put on the micro-grid (sample-side down) and pressed down (Fig. 3c). After the Si wafer was removed, some NWs with one end freely suspended and the other end embedded into the carbon conductive adhesive could be found (Fig. 3d–e).

**Fig. 4:**
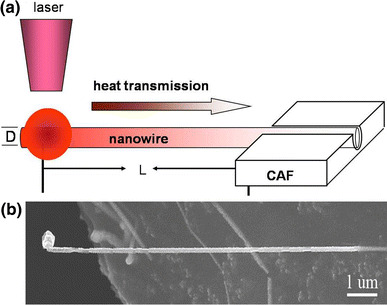
**a** The schematic diagram of thermal conductivity test; **b** The HRSEM image of the tested single NW

Figure 4a shows the schematic diagram of experimental setup for thermal conductivity measurement. The 514 nm Ar^+^ laser was focused with a 100× objective lens to a 1.5-µm diameter spot (Airy disk) and used for local heating of the NWs. In this process, the laser output power (*P*_0_) was continually increased until NWs were fused. As we all know, power distribution of laser follows Gaussian distribution, and the power of the Airy disk (*P*_A_) is 84 % of the total laser output power (*P*_0_) [23]. In addition, the diameter of the Airy disk (*D*_0_) and the laser output power (*P*_0_) are known. The laser power density was obtained (*ρ*). Then the laser-irradiated power on the NW surface (*P*_s_) was calculated using the power density integration. To the best of our knowledge, the energy of light has exponent-type inner absorptions with increasing propagation distance in media. The laser absorbing efficiency is given by *β* = e^−*αl*^, where 0 ≤ *l*≤*D* is the propagation distance of laser in the NW (*D*: diameter of NW) and *α* is absorption coefficients for 514 nm laser, about 3.29 × 10^7^ m^−1^ [24]. Based on the above data, the absorption efficiency can be calculated around 1. D. E. Aspnes et al. have reported that the reflectivity *γ* of InAs NW for 514 nm laser is 0.44 [24]. Combined with the radiant energy on the NW surface (*P*_s_), the *P*_abs_ can be calculated by *P*_abs _= *β*(1−γ)*P*_abs_.

## Results and Discussion

In this work, the thermal conductivity measurement was performed in ambient air. Therefore, the absorbed power (*P*_abs_) inside the NWs is equal to the sum of the intrinsic heat conduction power of NW ($\dot{Q}_{ci}$), the heat loss power through air conduction ($\dot{Q}_{ac}$), the heat convection loss power ($\dot{Q}_{c}$), and the radiation loss power ($\dot{Q}_{r}$), as follows:

1 $$ P_{{\rm abs}} = \dot{Q}_{ci} + \dot{Q}_{ac} + \dot{Q}_{c} + \dot{Q}_{r} $$

Considering the thermal conductivity of air (10^−2^ W m^−1 ^k^−1^) is much smaller than InAs NW (10^0^ W m^−1^ k^−1^), $\dot{Q}_{ac}$ is negligible [13, 25, 26]. $\dot{Q}_{c}$ is the convective heat loss, which is given by *A*_*s*_*h(T*_*1*_ – *T*_*2*_*)*, in which *A*_*s*_ is the superficial area of NW, 1 < *h*<10 is the convective heat transfer coefficient of air  [27], *T*_1_ and *T*_2_ are the NW surface temperature and near-surface air temperature, respectively. $\dot{Q}_{r}$ is the radiation loss, which can be obtained by *εA*_*s*_*σ* (*T*_1_^4^–*T*_2_^4^), where *ε* is emissivity (always less than 1) and *σ* = 5.67 × 10^−8^ W ^−2 ^K^−4^ is the Stefan–Boltzman constant.

Here, the maximum *h* (10), maximum *T*_1_ (melting of nanowire), minimal *T*_2_ (25°), and maximum *ε* (1) were employed to estimate the upper limit of $\dot{Q}_{c}$ and $\dot{Q}_{r}$. The $\dot{Q}_{c}^{\hbox{max} }$and $\dot{Q}_{r}^{\hbox{max} }$ are much less than that of *P*_abs_ (see Table 1). Therefore, the $\dot{Q}_{c}$ and $\dot{Q}_{r}$ are negligible, and the formula $\dot{Q}_{ci} = P_{{\rm abs}}$ can be conducted. A simple model to describe the heat conduction in one-dimensional nanostructures has been reported by Hsu et al. [28], which is

2 $$ P_{{\rm abs}} = \dot{Q}_{ci} = \Delta T/[L/(KA) + R_{c} ], $$

where *L* is the length of the suspended segment, *A* is the cross section area of NWs, and Δ*T* is the temperature difference between the melting point (*T*_m_) of NWs and contact points. On one hand, the melting point of NWs can be obtained according to the deposited temperature region of the NWs with different diameters. On the other hand, one end of the NWs embedded into the carbon conductive adhesive, which results in large area of heat dissipation. So the temperature of contact point is almost equal to the room temperature, and the contact thermal resistances (*R*_*c*_) between the NWs and the conductive adhesive can be neglected [28]. In this work, we measured the thermal conductivity of InAs NWs with different diameters using this laser heating method. These results are summarized in Table 1.

**Table 1:** Summary of the radius increase measurements of five suspended NWs

Sample	*L* (µm)	*D*(nm)	*T*m (±10 °C)	*P*_abs_ (μW)	$\dot{Q}_{c}^{\hbox{max} }$(μW)	$\dot{Q}_{r}^{\hbox{max} }$(μW)	*K*(W m^−1^ k^−1^)
1	9.8	107	405	2.23	0.0126	0.0050	6.40
2	10.2	110	407	2.37	0.0135	0.0053	6.66
3	10.3	130	420	4.30	0.0166	0.0074	8.45
4	10.5	170	447	9.29	0.0237	0.0127	10.19
5	10.0	189	459	12.75	0.0258	0.0149	10.48

**Fig. 5:**
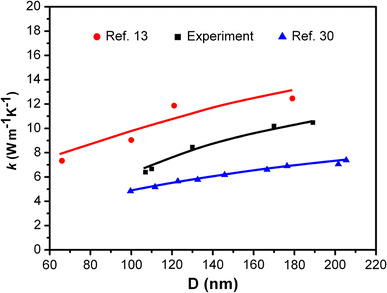
Thermal conductivity of the measured InAs NWs and Datum from previous results are also shown (Refs. [13] and [30]). (Color figure online)

The obtained thermal conductivities of NWs with different radius are shown in Fig. 5. Compared with the previous experimental results [13], the *k* values in air are slightly lower than those in vacuum. This might be due to the oxidation and the formation of amorphous arsenic induced by laser heating [10]. Moreover, the thermal conductivity presents monotonic decrease with decreasing diameters, which is ascribed to the enhanced phonon-boundary scattering [29]. In a solid nanowire, the boundary scattering is usually treated by Casimir limit [30], from which the effective phonon mean free path is given by $\bar{\lambda } = D$. Therefore, the phonon mean free path is reduced with decreasing of wire diameter, resulting in the reduction of thermal conductivity [29]. To gain a quantitative understanding of the diameter-dependent thermal transport in InAs NWs, the results of the thermal conductivity calculation as a function of NWs diameter by N. Mingo are also shown in Fig. 5 [30]. It can be seen that the calculated values are close to our experimental data, which further confirms that the decrease of thermal conductivity is due to the decrease of diameter.

## Conclusions

High-quality InAs NWs were synthesized via a simple and low-cost CVD method. XRD, HRTEM, in situ EDS, and selected area electron diffraction (SAED) confirmed the single-crystal quality of these InAs NWs. More importantly, the diameter-dependent thermal conductivity of InAs NWs in air was systematically studied by a home-made laser heating method. The measured thermal conductivity agrees well with the previously reported results. Based on the simple method, it may be helpful for further studying and evaluating thermoelectric properties of NWs.
